# 2905. Development of a Desirability of Outcome Ranking (DOOR) for Skin Infection Clinical Trials

**DOI:** 10.1093/ofid/ofad500.176

**Published:** 2023-11-27

**Authors:** Loren G Miller, Donna Phan Tran, Evelyn A Flores, Honghu Liu, Beverly A Weidmer, Deborah Kupferwasser, Mary G Boyle, Pluscedia Williams, Linyu Zhou, Yilan Huang, Scott R Evans, Stephanie A Fritz

**Affiliations:** Lundquist Institute at Harbor-UCLA Medical Center, Los Angeles, California; Division of Infectious Diseases, the Lundquist Institute at Harbor-UCLA Medical Center, Torrance, CA, Torrance, California; Division of Infectious Diseases, the Lundquist Institute at Harbor-UCLA Medical Center, Torrance, CA, Torrance, California; University of California, Los Angeles, School of Dentistry, Public Health and Medicine, Los Angeles CA, Los Angeles, California; RAND Corporation, Santa Monica, CA, Santa Monica, California; Division of Infectious Diseases, the Lundquist Institute at Harbor-UCLA Medical Center, Torrance, CA, Torrance, California; Washington University School of Medicine, St. Louis, Missouri; Charles R. Drew University, Los Angeles, CA, Los Angeles, California; Public and Population Health, UCLA School of Dentistry, Los Angeles, California; University of California, Los Angeles, Department of Biostatistics, Los Angeles CA, Los Angeles, California; Milken Institute School of Public Health, Rockville, Maryland; Washington University School of Medicine, St. Louis, Missouri

## Abstract

**Background:**

Infectious disease clinical trials treatment outcomes have almost exclusively been dichotomized into cure and failure. The novel Desirability of Outcome Ranking (DOOR) method uses a hierarchical ordinal outcome of intervention outcomes. DOOR measures are more sensitive for detection of differences between groups compared to dichotomous outcomes and thus can lead to dramatic reduction of the number of required trial participants. Additionally, the DOOR design addresses patient-centered outcomes that are ignored in traditional infectious diseases clinical trial outcomes. DOOR methodology has neither been used for skin and soft tissue infection (SSTI) clinical trial outcomes nor incorporated patients about their experiences with SSTIs.

**Methods:**

We conducted 6 focus groups of providers, patients, and parents at two medical centers in Los Angeles and St. Louis. Focus groups were composed of clinicians who treat SSTIs (n=2), patients who had SSTIs (n=3) and parents of children who had an SSTI (n=1). Focus group participants were given a series of SSTI clinical scenarios with various outcomes and asked to rank these 10 outcomes on a numerical scale from most to least desirable. We fitted 4 CART (classification and regression tree) models for overall, provider, patient and parent responders, respectively.

**Results:**

In total, we had 62 focus group participants (24 providers, 30 patients, and 8 parents). Overall, the most desirable outcome was no complications (rank value = 1.0), followed by rash or nausea (mean rank values = 4.9 - 5.1), diarrhea or recurrent infection (= 5.9), dizziness or stomach ache (= 6.1 - 6.2), recurrent drainage procedure or household transmission (= 7.0 - 7.2), and hospitalization (= 8.3). Least desirable outcomes differed between groups, with providers choosing hospitalization while patient/parents indicated household transmission. Variance in outcome desirability differed more widely between patients compared to providers. (Figure).

**Figure:**
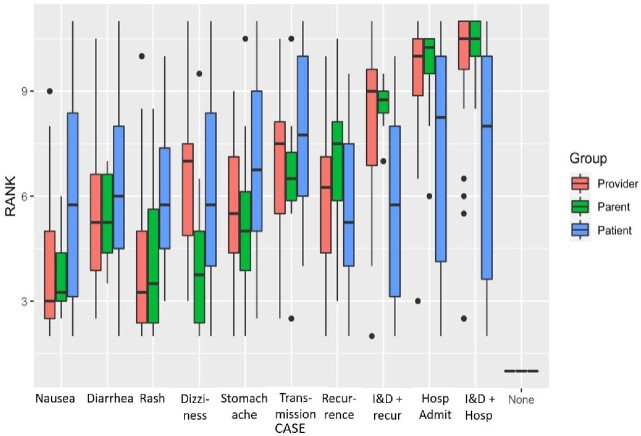

**Conclusion:**

We developed a DOOR outcome for SSTIs clinical trials that incorporates both provider and patient perspectives. This metric could be tested in clinical trials as a means to reduce sample size and to incorporate patient-focused outcomes.

**Disclosures:**

**Loren G. Miller, MD MPH**, ContraFect: Grant/Research Support|GSK: Grant/Research Support|Medline: Grant/Research Support|Merck: Grant/Research Support|Paratek: Grant/Research Support

